# Superconcentrated electrolytes for a high-voltage lithium-ion battery

**DOI:** 10.1038/ncomms12032

**Published:** 2016-06-29

**Authors:** Jianhui Wang, Yuki Yamada, Keitaro Sodeyama, Ching Hua Chiang, Yoshitaka Tateyama, Atsuo Yamada

**Affiliations:** 1 Department of Chemical System Engineering, University of Tokyo, 7-3-1, Hongo, Tokyo 113-8656, Japan; 2 Elements Strategy Initiative for Catalysts and Batteries, Kyoto University, 1-30, Goryo-Ohara, Kyoto 615-8245, Japan; 3 JST PRESTO, 4-1-8 Honcho Kawaguchi, Saitama 332-0012, Japan; 4 International Center for Materials Nanoarchitectonics, National Institute for Materials Science, 1-1, Namiki, Tsukuba 305-0044, Japan

## Abstract

Finding a viable electrolyte for next-generation 5 V-class lithium-ion batteries is of primary importance. A long-standing obstacle has been metal-ion dissolution at high voltages. The LiPF_6_ salt in conventional electrolytes is chemically unstable, which accelerates transition metal dissolution of the electrode material, yet beneficially suppresses oxidative dissolution of the aluminium current collector; replacing LiPF_6_ with more stable lithium salts may diminish transition metal dissolution but unfortunately encounters severe aluminium oxidation. Here we report an electrolyte design that can solve this dilemma. By mixing a stable lithium salt LiN(SO_2_F)_2_ with dimethyl carbonate solvent at extremely high concentrations, we obtain an unusual liquid showing a three-dimensional network of anions and solvent molecules that coordinate strongly to Li^+^ ions. This simple formulation of superconcentrated LiN(SO_2_F)_2_/dimethyl carbonate electrolyte inhibits the dissolution of both aluminium and transition metal at around 5 V, and realizes a high-voltage LiNi_0.5_Mn_1.5_O_4_/graphite battery that exhibits excellent cycling durability, high rate capability and enhanced safety.

## Abstract (web summary)

Electrode degradation due to metal-ion dissolution in conventional electrolyte hampers the performance of 5 V-class lithium ion batteries. Here, the authors employ a high concentration electrolyte to inhibit metal-ion dissolution and realize a stable high voltage LiNi_0.5_Mn_1.5_O_4_/graphite battery.

## Graphical abstract

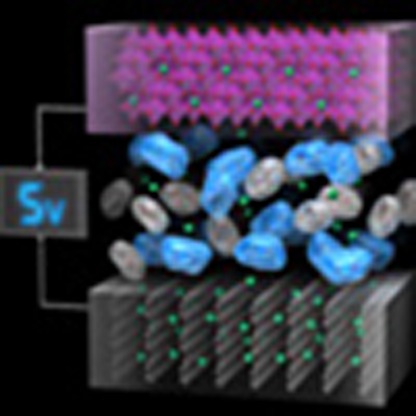

Lithium-ion batteries, having received great commercial success in the portable power source market, are being aimed for large-scale energy-storage application in electric vehicles^1^,^2^,^3^. To approach the high energy-density requirements for automobiles, a pragmatic approach is to elevate the operating voltage of batteries, from the present 4 V to around 5 V (refs 4, 5). This allows the direct application of the mature fabrication technology of 4 V-class lithium-ion batteries, the well-developed negative electrodes (for example, graphite and graphite/silicon), and high-voltage positive electrodes (for example, spinel LiNi_0.5_Mn_1.5_O_4_ and some layered oxides). However, new challenges—which mainly arise from the electrolyte—hinder the practical application of the next-generation 5 V-class battery.

One major problem is metal dissolution from the positive electrode at high voltages, which poses a serious dilemma in designing an electrolyte. In state-of-the-art lithium-ion electrolyte, chemically unstable LiPF_6_ is an essential component to suppress anodic (oxidative) dissolution of an aluminium current collector because its hydrolysis product of hydrofluoric acid (HF) contributes to an insoluble AlF_3_ passivation film^6^,^7^. However, the generated HF accelerates the dissolution of transition metals from the active electrode materials, which causes severe capacity decay upon cycling, especially at high voltages and elevated temperatures^8^,^9^. Using LiNi_0.5_Mn_1.5_O_4_ as an example, the dissolved Mn^2+^ and Ni^2+^ ions, albeit <1% of the total amount, deposit on the surface of the graphite negative electrode, which thicken the solid electrolyte interphase (SEI) by catalysing the reductive decomposition of the electrolyte, and consume the limited lithium reserve in the battery to result in a >50% capacity loss in 100 charge/discharge cycles^10^,^11^. Diversified functional additives and/or alternative solvents have been explored^12^,^13^,^14^,^15^,^16^,^17^ but improvements are still unsatisfactory. Efforts have tried more stable salts (less tendency to generate HF) to replace LiPF_6_, such as lithium perfluorosulfonylamide (shortened to ‘amide')^18^. However, the chemically stable amide does not participate in the reaction with Al to form a stable passivation film, thus causing severe anodic dissolution of the Al current collector at >4 V (refs 19, 20, 21, 22). As a result, it remains a dilemma for electrolyte design to suppress both the Al dissolution (requiring an unstable salt) and transition metal dissolution (avoiding an unstable salt). Recently, increasing the concentration of amide salts was reported to alleviate anodic Al dissolution^23^,^24^,^25^, but the operating voltage of a half-cell is still limited below 4.3 V, presumably owing to some or all of the following reasons: insufficient salt concentration^23^, too low ionic conductivity^24^ and too low oxidative stability of the solvent^26^.

In this work, we report an electrolyte system to resolve the dilemma. We select stable yet dissociative lithium bis(fluorosulfonyl)amide (LiFSA) as the salt and oxidation-stable carbonate esters as the solvent. We demonstrate an unusual liquid with a peculiar three-dimensional structural network obtained at extremely high salt concentrations. The superconcentrated electrolyte not only effectively suppresses the anodic Al dissolution but also remarkably inhibits the transition metal dissolution and, thus, realizes a safe, stable and fast-rate high-voltage LiNi_0.5_Mn_1.5_O_4_|graphite battery.

## Results

### Physicochemical properties

**Figure 1:**
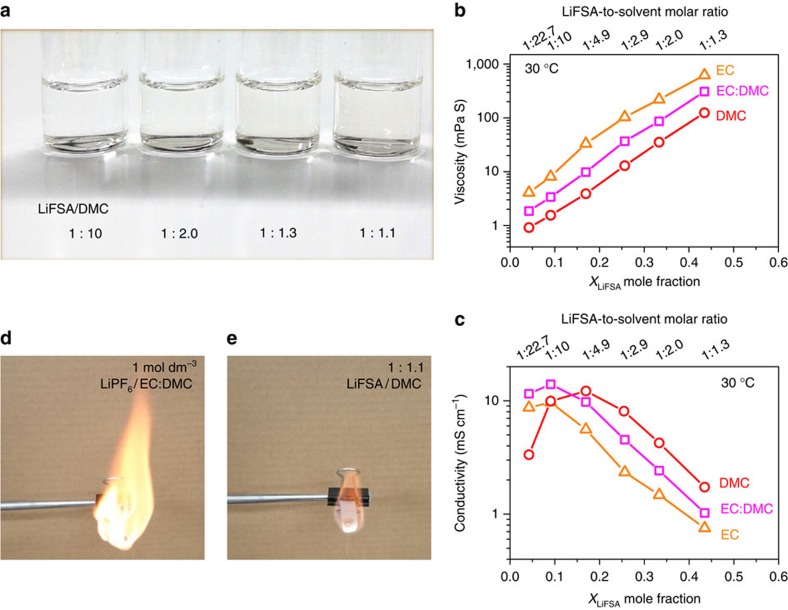
Physicochemical properties dependent on solution concentration. (**a**) Images of various salt-to-solvent molar ratios of LiFSA/DMC solutions. Viscosity (**b**) and ionic conductivity (**c**) for solutions of LiFSA in DMC, EC and EC:DMC (1:1 by mol.) at 30 °C. The *X*_LiFSA_ mole fraction is the molar amount of LiFSA salt divided by the total molar amount of the salt and solvents. The LiFSA-to-solvent molar ratios of the solutions are shown on the upper axis. (**d**) Flame tests of a commercial dilute electrolyte of 1.0 mol dm^−3^ LiPF_6_/EC:DMC (1:1 by vol.) and (**e**) the lab-made superconcentrated electrolyte of 1:1.1 LiFSA/DMC.

LiFSA salt was dissolved at various concentrations into three different carbonate ester solvents: dimethyl carbonate (DMC), ethylene carbonate (EC) and mixed EC:DMC. All the mixtures are transparent liquids at room temperature (see Fig. 1a as an example). Their basic physicochemical properties are presented in Supplementary Table 1. Figure 1b shows their viscosity as a function of salt concentration. Independent of the solvents used, the viscosity increases exponentially with increasing the LiFSA mole fraction (*X*_LiFSA_). Among the three groups of solutions, the group with DMC as the solvent shows the lowest viscosity because pure DMC has a lower viscosity than pure EC or mixed EC:DMC. For electrolytes with similar solvation radiuses of mobile ions, the ionic conductivity is proportional to the number of mobile ions and inversely proportional to the viscosity of the medium^18^. As shown in Fig. 1c, at dilute concentrations of *X*_LiFSA_<0.14 (below 1.5 mol dm^−3^), the use of the EC:DMC mixture shows the highest ionic conductivity owing to a synergistic effect: the high-dielectric-constant EC increases the mobile ion number by promoting salt dissociation; the low-viscosity DMC increases the ion mobility by decreasing the solution viscosity. This is why the mixed solvents of EC and linear carbonates are generally adopted in conventional electrolytes of the lithium-ion battery^18^. However, when *X*_LiFSA_ is above 0.14, the solution with DMC as the sole solvent shows an even higher ionic conductivity than that with EC:DMC, which should result from the much lower viscosity of the former at high concentrations. This result suggests that the viscosity becomes the decisive factor on the ionic conductivity for a concentrated solution, wherein intensive ionic association exists independent of the solvents used, showing a distinct departure from the conventional electrolyte design strategy on the basis of dilute concentrations. For the LiFSA/DMC solution, a commercially acceptable ionic conductivity of 1.12 mS cm^−1^ (30 °C) is obtained even at a ‘super-high' concentration with salt-to-solvent molar ratio of 1:1.1 despite a high viscosity of 238.9 mPa s. Although the ionic conductivity is lower than that of the commercial dilute electrolyte, it does not compromise the rate capability of the battery (shown later).

On the other hand, the drawbacks of the high volatility and high flammability of linear carbonate solvents can be overcome to a large degree owing to the much lower content of organic solvents in the concentrated solutions. Thermogravimetry measurements (Supplementary Fig. 1) show that the weight loss of the superconcentrated 1:1.1 LiFSA/DMC solution is only 1.5 wt% after elevating the temperature to 100 °C, which is considerably lower than those of a dilute 1:10.8 LiFSA/DMC solution (65.5 wt%, corresponding to 1.0 mol dm^−3^) and a commercial electrolyte (28.7 wt%). As demonstrated in the flame tests (Fig. 1d,e), the 1:1.1 LiFSA/DMC solution does not burn as fiercely as the commercial dilute electrolyte. The superior thermal stability and flame retardant ability of the concentrated electrolytes contribute to the remarkably improved safety properties as compared with the dilute electrolytes.

### Reversible reaction of a 5 V-class electrode

Anodic dissolution of the Al current collector and/or oxidative decomposition of solvent may be encountered in the high-voltage application of amide-based electrolytes. To exclude the possible influence of the anodic Al dissolution, we initially used platinum foil as the current collector for the LiNi_0.5_Mn_1.5_O_4_ electrode in a three-electrode cell (Supplementary Fig. 2). The results showed that both dilute (1:10.8) and superconcentrated (1:1.1) LiFSA/DMC electrolytes enabled a reversible Li^+^ de-intercalation/intercalation on the LiNi_0.5_Mn_1.5_O_4_|Pt electrode, indicating a reasonably good compatibility between the present electrolyte system and LiNi_0.5_Mn_1.5_O_4_ material at ∼5 V.

**Figure 2:**
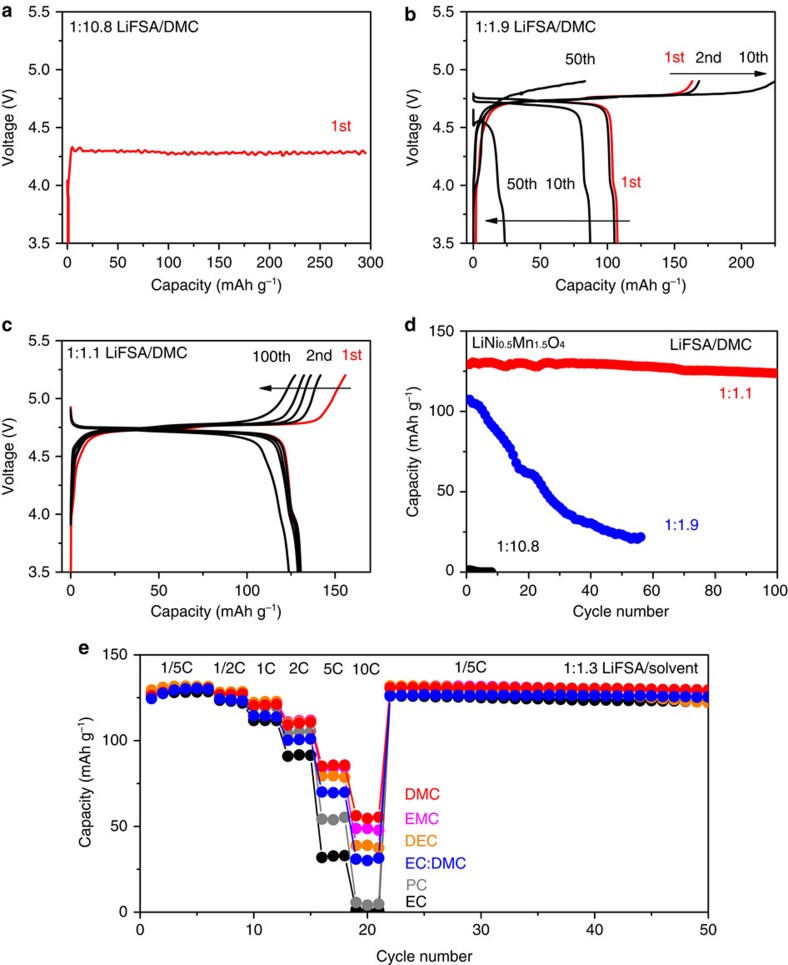
Performance of 5 V-class LiNi_0.5_Mn_1.5_O_4_ electrode in a half-cell. Charge–discharge voltage curves of LiNi_0.5_Mn_1.5_O_4_|lithium metal half-cells using (**a**) dilute 1:10.8, (**b**) moderately concentrated 1:1.9 and (**c**) superconcentrated 1:1.1 LiFSA/DMC electrolytes at a C/5 rate. Some/all curves of 1st, 2nd, 10th, 50th and 100th cycles are shown. (**d**) Discharge (Li^+^ intercalation) capacity retention of the half-cells using different concentrations of LiFSA/DMC electrolytes at a C/5 rate. (**e**) Rate capacity and subsequent cycling retention of the half-cells using 1:1.3 LiFSA-based electrolytes with different carbonate solvents. Charge–discharge tests were conducted at 25 °C with a cutoff voltage of 3.5–4.9 V and a maximum-time restriction of 10 h except for that using the 1:1.1 LiFSA/DMC electrolyte whose cutoff voltage was 3.5–5.2 V. The 1C-rate corresponds to 147 mA g^−1^ on the weight basis of the LiNi_0.5_Mn_1.5_O_4_ electrode.

However, when applied in a coin cell using the conventional Al current collector, low concentrations of LiFSA/DMC electrolytes encountered problems, confirming the critical drawback of anodic Al dissolution for the amide-based electrolytes. As shown in Fig. 2a, the first charge on the LiNi_0.5_Mn_1.5_O_4_|Al electrode is impossible in the dilute 1:10.8 LiFSA/DMC electrolyte owing to the continuous Al dissolution at 4.3 V. In the concentrated 1:1.9 LiFSA/DMC electrolyte (Fig. 2b), the charge/discharge cycling becomes possible up to the cutoff voltage of 4.9 V, but the large irreversible capacity indicates the parasitic Al dissolution remains. The Al dissolution subsequently deteriorates the electrical contacts between the LiNi_0.5_Mn_1.5_O_4_ material and the Al current collector, and results in a fast capacity decay (Fig. 2d). Actually, the poor cycling performance on the LiNi_0.5_Mn_1.5_O_4_ electrode was generally observed in other concentrated 1:2 LiFSA/carbonate ester electrolytes, such as LiFSA in EC, propylene carbonate (PC), ethyl methyl carbonate (EMC) and diethyl carbonate (DEC; Supplementary Fig. 3), indicating this concentration is not sufficient to fully inhibit Al dissolution at 5 V.

In contrast, the superconcentrated 1:1.1 LiFSA/DMC electrolyte enables a reversible Li^+^ de-intercalation/intercalation reaction on the LiNi_0.5_Mn_1.5_O_4_ electrode even at a high voltage of 5.2 V (Fig. 2c). In a charge/discharge cycling test, the capacity retention after 100 cycles was over 95% (Fig. 2d), and the coulombic efficiency was close to 100% (Supplementary Fig. 4), evidencing an effective inhibition of anodic Al dissolution. Similarly, using the super-high concentration of 1:1.3, all the LiFSA/carbonate electrolytes enabled stable charging/discharging cycling of the LiNi_0.5_Mn_1.5_O_4_ electrode (see Fig. 2e for example). Especially, the electrolytes using low-dielectric-constant and low-viscosity linear carbonate solvents (for example, DMC, EMC and DEC) showed a faster rate capability as compared with those using high-dielectric-constant and high-viscosity cyclic carbonate solvents (for example, EC, PC and their corresponding mixtures), which is at least partly owing to the much higher ionic conductivity of the former. These results demonstrate that the salt-superconcentrated strategy is a simple, effective and fruitful approach to various safe and stable high-voltage electrolytes. To the best of our knowledge, this is the first time that stable and fast charge/discharge cycling of a 5 V-class electrode using amide salt-based organic electrolytes has been achieved.

**Figure 3:**
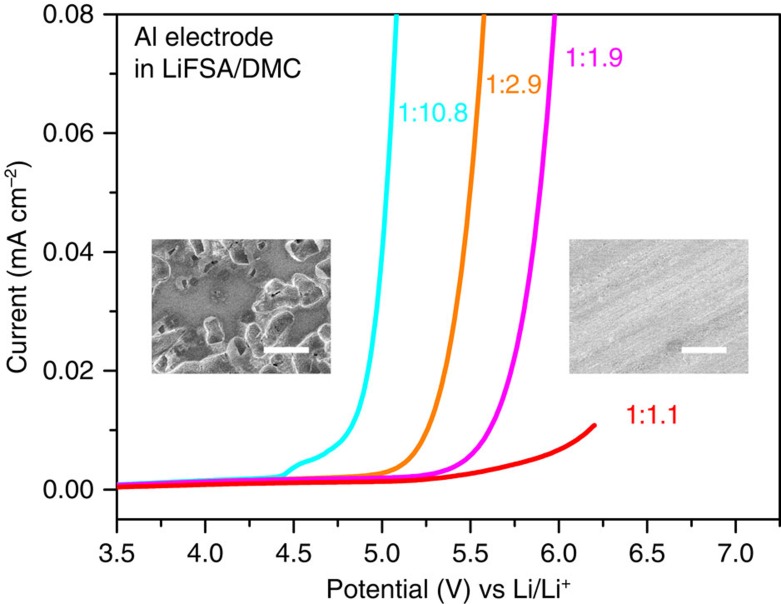
Oxidation stability of an aluminium electrode. LSV of an aluminium electrode in various concentrations of LiFSA/DMC electrolytes in a three-electrode cell. The scan rate was 1.0 mV s^−1^. The insets are scanning electron microscopy images of the Al surface polarized in the dilute 1:10.8 (left of panel) and superconcentrated 1:1.1 (right of panel) electrolytes. Many corroding pits cover the surface of the Al electrode polarized in the dilute electrolyte, showing a severe anodic Al dissolution. In contrast, no corroding pits appear on the surface of the Al electrode polarized in the superconcentrated electrolyte, indicating a good inhibition of anodic Al dissolution. The white scale bar represents 20 μm.

The progressive inhibition of anodic Al dissolution with increasing salt concentration is further proved by linear sweep voltammetry (LSV) of an Al electrode and the subsequent scanning electron microscopy observation on the polarized Al surface (see Fig. 3 for details). This notable concentration effect was recently reported but with a debate on whether a stable surface film on Al (ref. 23) or the elimination of uncoordinated (free) solvents of electrolyte^24^,^25^ plays the key role. We conducted a surface analysis of the Al electrodes polarized in various concentrations of LiFSA/DMC electrolytes by X-ray photoemission spectroscopy (XPS) as well as a comparative LSV study between fresh and polarized Al electrodes (see Supplementary Figs 5 and 6 for details). We were unable to obtain any essential evidence to support the existence of a stronger surface film generated in the concentrated electrolyte. Instead, we found that the LiFSA salt readily decomposes and produces LiF upon Ar^+^ etching (Supplementary Fig. 7). The previous observation in ref. 23—a much thicker surface film of LiF produced in a higher concentration of electrolyte—is likely to arise from the decomposition of un-rinsed amide salt induced by Ar^+^ etching in the XPS measurement.

**Figure 4:**
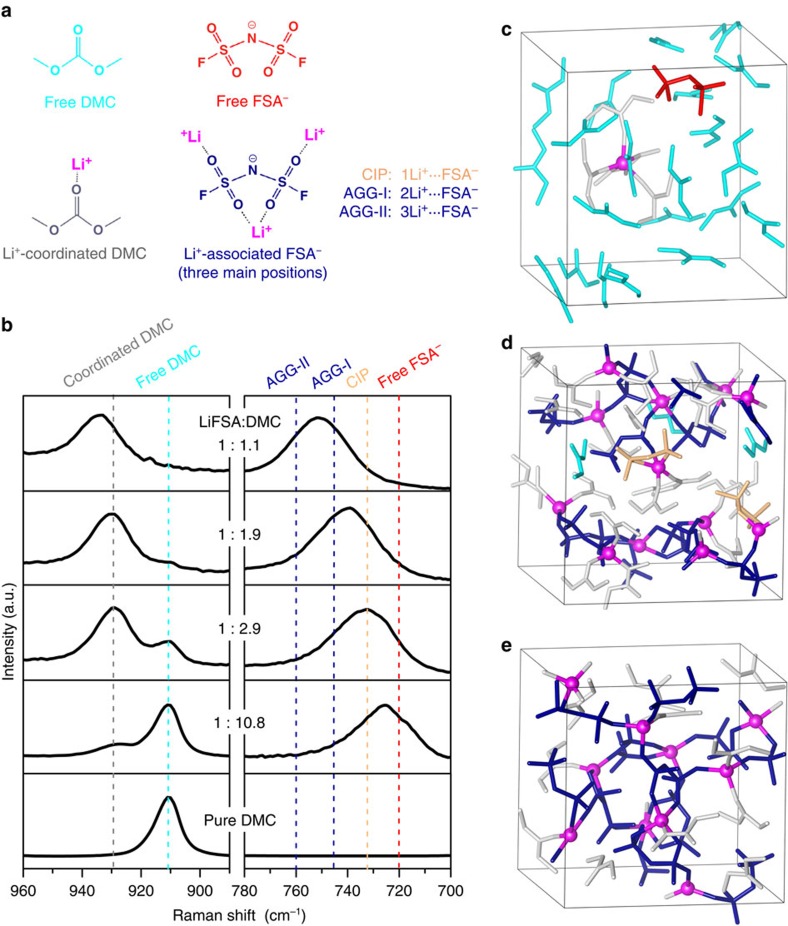
Li salt−solvent coordination structure dependent on salt concentration. (**a**) The several main species in the LiFSA/DMC solutions. (**b**) Raman spectra of LiFSA/DMC solutions with various salt-to-solvent molar ratios in the range of 890–900 cm^−1^ (O-CH_3_ stretching mode of the DMC solvent) and 700–780 cm^−1^ (S-N stretching mode of the FSA^−^ anion). Snapshots of typical equilibrium trajectories obtained by DFT-MD simulations: (**c**) dilute solution (1 LiFSA/25 DMC, <1 mol dm^−3^), (**d**) moderately concentrated solution (12 LiFSA/24 DMC, ca. 4 mol dm^−3^), and (**e**) superconcentrated solution (10 LiFSA/11 DMC, ca. 5.5 mol dm^−3^). The coordination of Li^+^−DMC and Li^+^−FSA^−^ is supposed to build up when the involved atoms locate within 2.5 Å from Li^+^. The coordination numbers of solvents and anions to Li^+^ are shown in Supplementary Fig. 8. Li cations are marked in purple. Free and coordinated DMC molecules are marked in light blue and grey, respectively. Free, CIP and AGG states of FSA^−^ anions are marked in red, orange and dark blue, respectively. Hydrogen atoms are not shown.

We now study the solution structure of the electrolytes using Raman spectroscopy observation and density functional theory molecular dynamics simulation (DFT-MD). As shown in the Raman spectra (Fig. 4b left), a free DMC molecule exhibits an O-CH_3_ stretching vibration band at 910 cm^−1^ (ref. 27). This band shifts up to 930–935 cm^−1^ when DMC participates in Li^+^ solvation. In dilute 1:10.8 LiFSA/DMC, the majority of DMC molecules exist in a free state because the solvent-to-salt molar ratio (10.8) is much larger than a typical four- or fivefold coordination of Li^+^ in aprotic solvents. As the LiFSA concentration increases, the population of free DMC decreases and that of Li^+^-coordinated DMC increases; the Li^+^-FSA^−^ association simultaneously intensifies through the formation of contact ion pairs (CIPs, FSA^−^ coordinating to one Li^+^) and aggregate clusters (AGGs, FSA^−^ coordinating to two or more Li^+^). The latter is evidenced from a remarkable upshift of the FSA^−^ band (700–780 cm^−1^, Fig. 4b right), which is typically observed in the amide-based concentrated solutions^24^,^25^,^28^,^29^,^31^. For the moderately concentrated 1:2 LiFSA/DMC solution, the Raman band corresponding to free DMC is remarkably weakened, suggesting that the majority of DMC are solvating to Li^+^. This is consistent with the DFT-MD simulation, which shows ca. 90% DMC are coordinating to Li^+^ with the rest as free solvent (marked as light blue in Fig. 4d). Moreover, the simulation illustrates that all FSA^−^ anions are coordinating to Li^+^ with ca. 20% as CIPs and ca. 80% as AGGs (marked as orange and dark blue in Fig. 4d, respectively). The coordination environment is shown in Supplementary Fig. 8. For the superconcentrated 1:1.1 LiFSA/DMC solution, both DMC and FSA^−^ bands further upshift substantially, indicating both Li^+^-DMC and Li^+^-FSA^−^ interactions enhanced compared with those in 1:2 LiFSA/DMC. The DFT-MD simulation reveals that all DMC molecules, together with all FSA^−^ anions, are coordinating to Li^+^ (no free solvent or anion). Interestingly, besides oxygen, significant amount of nitrogen on FSA^−^ anions also participate in the coordination with Li^+^, which is hardly observed at the lower concentrations. The contribution of nitrogen coordinating to Li^+^ is shown in Supplementary Fig. 8. More importantly, almost all FSA^−^ anions remain in AGG states during the whole DFT-MD simulation time (0.1 fs × 100,000 steps), and a CIP state is rarely observed, demonstrating the unusual solution structural feature with AGG clusters as the predominant components in the superconcentrated LiFSA/DMC solution. It is noteworthy that each FSA^−^ anion coordinates to 2–3 Li^+^ and each Li^+^ is coordinated by 2–3 FSA^−^ in 1:1.1 LiFSA/DMC. Hence, FSA^−^ anions in the superconcentrated solution connect with each other through the intensive association with Li^+^, leading to a reinforced three-dimensional network (shown in Fig. 4e). This feature is different from the less concentrated solutions, wherein significant amount of CIPs and free solvents divide the solution structure into relatively small-size parts.

Generally, the anodic metal dissolution requires three steps: first, oxidation of the metal to a metal cation; second, coordination of the metal cation by solvents or anions; and finally, the diffusion of the solvated metal cation to the bulk electrolyte^32^. At high voltages of ∼5 V, the first step proceeds more rapidly and extensively than at the conventional operating voltage of 4 V. Thereby, the subsequent coordination and diffusion must be strongly inhibited by the nature of electrolyte solutions to suppress the metal ion dissolution. In the moderately concentrated 1:2 LiFSA/carbonate electrolytes, the presence of significant free solvents and CIPs (with two or more coordination sites remaining vacant) could coordinate to Al cations and fail to inhibit Al dissolution completely at 5 V. In contrast, the superconcentrated 1:1.1 LiFSA/DMC electrolyte effectively inhibits Al dissolution even over 5 V, which can be ascribed to its peculiar AGGs-predominant solution structure: (i) all DMC solvents and all FSA^−^ anions strongly coordinate to Li^+^ cations and thus have a much lower probability of coordinating to other metal cations; (ii) the resulting reinforced three-dimensional network further retards the diffusion rate of the metal cations, particularly, those with multiple charge.

### Stable cycling of a 4.6 V LiNi_0.5_Mn_1.5_O_4_|graphite battery

**Figure 5:**
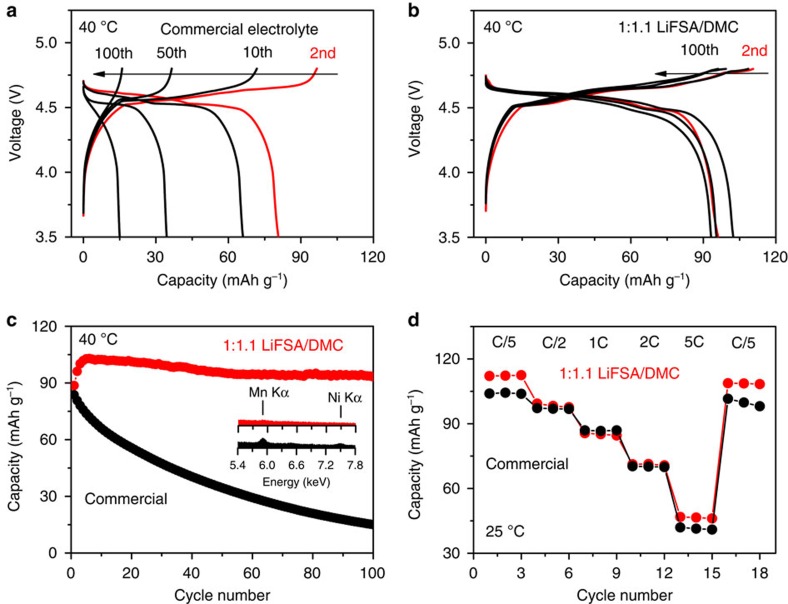
Performance of a high-voltage LiNi_0.5_Mn_1.5_O_4_|natural graphite battery. Charge–discharge voltage curves of LiNi_0.5_Mn_1.5_O_4_|graphite full cells using (**a**) a commercial 1.0 mol dm^−3^ LiPF_6_/EC:DMC (1:1 by vol.) electrolyte and (**b**) lab-made superconcentrated 1:1.1 LiFSA/DMC electrolyte at a C/5 rate and 40 °C. The curves of 2nd, 10th, 50th and 100th cycle are shown. (**c**) Discharge capacity retention of the full cells at a C/5 rate. The inset shows EDS spectra on the graphite electrode surface (200 × 200 μm^2^ area) after 8-day cycling tests, which is equivalent to the operating time of 100 and 20 cycles for the battery using the commercial and superconcentrated electrolytes, respectively. (**d**) Discharge capacity of the full cell at various C-rates and 25 °C. All charge-discharge cycling tests were conducted with a cutoff voltage of 3.5–4.8 V. 1C-rate corresponds to 147 mA g^−1^ on the weight basis of the LiNi_0.5_Mn_1.5_O_4_ electrode.

In addition to the excellent performance achieved on the LiNi_0.5_Mn_1.5_O_4_-positive electrode, the superconcentrated 1:1.1 LiFSA/DMC electrolyte also realized ultra-stable charge/discharge cycling on the natural graphite-negative electrode despite the absence of conventional SEI-forming agent of EC (Supplementary Fig. 9): the application in a graphite|Li half-cell exhibits a capacity retention of 99.6% after 100 cycles with coulombic efficiency of ∼99.8%, and with rate capability comparable with that using a commercial dilute electrolyte. Accordingly, the superconcentrated electrolyte was further applied in the high-voltage LiNi_0.5_Mn_1.5_O_4_|graphite full cell, a much harsher condition than in the half-cell, because the active lithium resource is limited and a new underlying problem arises from the transition metal dissolution from the LiNi_0.5_Mn_1.5_O_4_ especially at high voltages and elevated temperatures. Figure 5a,b shows charge/discharge voltage profiles of LiNi_0.5_Mn_1.5_O_4_|natural graphite full cells at 40 °C using a state-of-the-art commercial electrolyte and the lab-made superconcentrated electrolyte, respectively. The cell with the commercial electrolyte suffers from a severe capacity decay during cycling, that is, only 18% of the initial capacity left after 100 cycles (Fig. 5a,c), which is consistent with previous reports^11^,^33^. In contrast, the capacity retention using the 1:1.1 LiFSA/DMC electrolyte is over 90% after 100 cycles, exhibiting a remarkably improved cycling durability (Fig. 5b,c and Supplementary Figs 10 and 11). Notably, the superiority of the superconcentrated electrolyte becomes even more marked at 55 °C (Supplementary Fig. 12). It is generally accepted that the poor cycling performance of the LiNi_0.5_Mn_1.5_O_4_|graphite battery originates from the dissolution of transition metals from LiNi_0.5_Mn_1.5_O_4_ into the electrolyte, as introduced at the beginning of this article. Indeed, energy dispersive X-ray spectroscopy (EDS) observation shows a much lower content of Mn and Ni on the graphite electrode of the full cell cycled in the superconcentrated electrolyte than that in the commercial electrolyte, which provides evidence for the effective inhibition of transition metal dissolution in the former. There are two main reasons for the improved performance: (i) LiFSA is less reactive to produce HF as compared with LiPF_6_, which alleviates the corrosion of LiNi_0.5_Mn_1.5_O_4_ and, thus, reduces the formation of soluble Mn^2+^ and Ni^2+^; (ii) even if some Mn^2+^ and Ni^2+^ are formed on the surface of LiNi_0.5_Mn_1.5_O_4_, they can hardly dissolve in and transport through the AGGs-predominant superconcentrated electrolyte owing to the same functional manner for the inhibition of Al dissolution. Moreover, it is worth noting that the rate performance of the full cell using the superconcentrated 1:1.1 LiFSA/DMC is comparable with that using the commercial electrolyte (Fig. 5d), although the former shows an ionic conductivity one-order lower than the latter. The mechanistic understanding on the high-rate capability of the superconcentrated electrolyte is underway in our laboratory. To the best of our knowledge, this is the first case that, an electrolyte with such an ultra-simple formulation—a single salt and a single solvent without any additive—realizes stable cycling of a high-voltage lithium-ion battery.

## Discussion

The conventional dilute LiPF_6_/EC-based electrolytes have dominated the electrolyte market of 4 V-class lithium-ion batteries over the past 25 years; however, they show difficulties in satisfying the requirements of next-generation 5 V-class batteries in terms of both safety and stability. Our work demonstrates a number of electrolytes with a reinforced three-dimensional network that are obtained by simple mixing of a stable salt with a conventional carbonate solvent at ‘super-high' concentrations. Owing to its peculiar structural characteristics, the superconcentrated electrolytes overcomes the longstanding challenge faced by the unstable LiPF_6_-based electrolytes at high voltages (passivating the Al current collector versus accelerating the transition metal dissolution of the active material), thus, enables a stable operation of a 5 V-class battery. Emphasis is on the fact that the peculiar solution structure and functionalities are unique to such super-high concentrations (solvent/salt≈1.1), and cannot be achieved in moderately high concentrations (solvent/salt>1.8) as in previous reports^24^,^30^,^31^,^34^,^35^. Besides the positive electrode side, the superconcentrated electrolytes also show a good compatibility with the natural graphite-negative electrode even in the absence of EC. It breaks through the limitation of a general requirement of EC for a SEI formation for a lithium-ion electrolyte, and diversifies the electrolyte formulation towards various EC-free electrolytes. Different from the conventional electrolyte design that requires a high-dielectric-constant (usually high-viscosity) solvent, the superconcentrated electrolyte prefers a low-viscosity solvent. Although the ionic conductivity of the superconcentrated electrolyte is lower than that of the conventional dilute electrolyte, it does not necessarily compromise the rate capability of the battery. Clarification of the corresponding mechanism would be enlightening for developing novel high-power batteries. Furthermore, the superconcentrated electrolytes show superior thermal stability and flame retardant ability, alleviating the safety risk for a high-voltage battery using conventional dilute electrolytes. Finally, it is noteworthy that our reported superconcentrated electrolytes do not contain any additives, signifying the potential to further enhance the performance. These desirable features above outperform the conventional dilute electrolytes; meanwhile, the wide-temperature window of the liquid state (ensuring a good contact with the electrode materials), as well as the convenience of the approach, surpass the solid-state electrolytes. Therefore, the superconcentrated electrolytes might offer opportunities to build safe and stable high-voltage batteries that are not limited to the lithium-ion.

## Methods

### Preparation of electrolytes and electrodes

LiFSA (Nippon shokubai) and all solvents (DMC, DEC, EMC, EC and PC, Kishida Chemical Co. Ltd) were lithium battery grade and used without purification. Electrolyte solutions were prepared by mixing a given amount of LiFSA with solvents in an Ar-filled glove box. The commercial electrolyte of 1.0 mol dm^−3^ LiPF_6_/EC:DMC (1:1 by vol) was purchased from Kishida Chemical Co. Ltd and used as the reference. Both the lab-made LiFSA-based electrolytes and as-received commercial LiPF_6_-based electrolyte were dried by molecular sieve before tests. The water content was less than 2 p.p.m., as detected by a coulometric Karl Fischer Titrator.

The electrodes were fabricated by first well mixing the active materials of LiNi_0.5_Mn_1.5_O_4_ (Hosen Corp., mean particle size 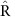
=5 μm, no surface treatment) and natural graphite (SEC Carbon Ltd., 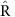
=10 μm), polyvinylidene difluoride (PVdF) and/or Denka black (AB, HS-100) in *N*-methylpyrrolidone with weight ratios of 80:10:10 (LiNi_0.5_Mn_1.5_O_4_:PVdF:AB) and 90:10 (graphite:PVdF). The resultant slurry was cast on the Al or Pt foil (20 μm thickness) for the LiNi_0.5_Mn_1.5_O_4_ electrode and on the Cu foil (10 μm thickness) for the graphite electrode using a 50 μm doctor blade. All those electrodes were dried at 120 °C under vacuum for 12 h. The active material mass loading was 0.7–2 mg cm^−2^ with a thickness of ∼15–20 μm, unless otherwise mentioned. The use of relatively low mass loading was to spotlight the critical issue of anodic Al dissolution as the content ratio of metallic Al components (Al current collector and Al positive cap) to the active electrode material becomes much higher in a coin cell. Nevertheless, thick electrodes with a high mass loading of ∼10 mg cm^−2^ were also tested. The results are shown in Supplementary Figs 11 and 12.

### Electrochemical measurements

LSV was performed by VMP-3 (BioLogic) in a beaker cell with an Al belt (1 × 4 cm^2^, 0.6 cm soaked in the electrolyte) as a working electrode and lithium metal as the reference and counter electrodes (shown in Supplementary Fig. 5 inset). LiNi_0.5_Mn_1.5_O_4_|Li half-cells and LiNi_0.5_Mn_1.5_O_4_|graphite full cells were assembled in the standard 2032-type coin cell hardware in an Ar-filled glove box. A combined separator, composed of cellulose separator (Nippon Kodoshi, placed on the positive electrode side) and glass fibre separator (Advantec GB-50, placed on the negative electrode side), was used. The amount of electrolyte in a coin cell was ca 160 μl to fully wet the separators and electrodes. In the full cells, the weight ratio of LiNi_0.5_Mn_1.5_O_4_:graphite was ∼2:1, which corresponds to ∼1:1.3 of their theoretical capacity ratio. Galvanostatic charge/discharge cycling and rate capability tests were conducted on a charge/discharge unit (TOSCAT). Charge and discharge were conducted at the same C-rate without using a constant-voltage mode at both ends of the charge and discharge.

### Characterization

The density and viscosity of solution samples were evaluated with a DMA 35 density meter and a Lovis 2000 M viscometer, respectively. The ionic conductivity was measured by AC impedance spectroscopy at 1 kHz (Solartron 147055BEC) in a symmetric cell (Pt|electrolyte|Pt). The flammability was tested on an electrolyte-soaked glass fibre filter (Advantec GB-100).

The solution structure was studied by a Raman spectroscopy with an exciting laser of 514 nm (NRS-5100). The samples were sealed in a quartz cell in the glove box to avoid any contamination from the air.

The morphology of Al electrodes after LSV tests were observed by a field-emission scanning electron microscopy at 2.0 kV. The transition metals deposited on the graphite electrode in the LiNi_0.5_Mn_1.5_O_4_|graphite full cells after charge/discharge cycling were estimated by an EDS. The cells were disassembled in the glove box. The obtained electrodes were rinsed in DMC and dried in the glove box. The sample was exposed in air for <1 min at sample loading.

The experimental details for thermogravimetric analysis and XPS measurements are shown in Supplementary Fig. 1 legend and Supplementary Methods, respectively.

### Simulations

Car-Parrinello type DFT-MD simulations were carried out using CPMD code^36^. LiFSA/DMC solutions with salt-to-solvent molar ratios of 1:25, 1:2 and 1:1.1 were calculated in cubic supercells with 15.05, 17.03 and 14.34 Å linear dimensions, respectively. A fictitious electric mass of 500 a.u. and a time step of 4 a.u. (0.10 fs) were chosen. The temperature was controlled using a Nosé thermostat with a target temperature of 30 °C. After 5 ps equilibration steps, statistical averages were computed from trajectories of at least 10 ps in length. The electronic wave function was quenched to the Born-Oppenheimer surface approximately every 1 ps to maintain adiabaticity. The energy cutoff of the plane wave basis is set to 90 Ry. Goedecker–Teter–Hutter type norm-conserving pseudopotentials for C, H, O, N, S, F and Li were used^37^.

### Data availability

The data that support the findings of this study are available from the corresponding author upon request.

## Additional information

**How to cite this article:** Wang, J. *et al*. Superconcentrated electrolytes for a high-voltage lithium-ion battery. *Nat. Commun.* 7:12032 doi: 10.1038/ncomms12032 (2016).

## Supplementary Material

Supplementary Information Supplementary Figures 1-12, Supplementary Table 1 and Supplementary Methods.
