# Supplementary material for: Superconcentrated electrolytes for a high-voltage lithium-ion battery
Source: Nat Commun. 2016 Jun 29;7:12032. doi: 10.1038/ncomms12032 (PMC4931331; doi:10.1038/ncomms12032)
Supplement: Supplementary Information — Supplementary Figures 1-12, Supplementary Table 1 and Supplementary Methods. [file ncomms12032-s1.pdf]

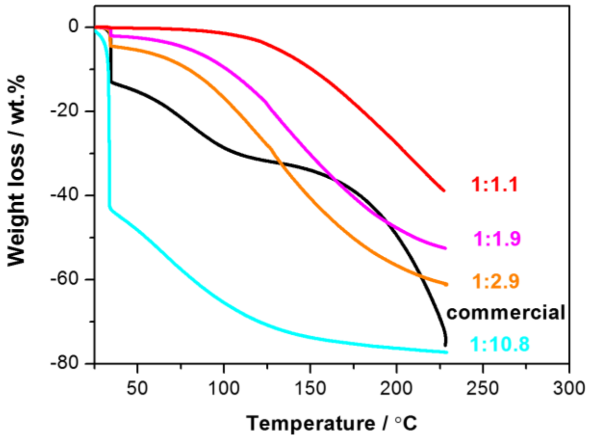

Supplementary Figure 1 Comparison of thermal stability of dilute and concentrated electrolytes. Weight loss of a commercial electrolyte (1.0 mol dm^{-3} $LiPF_6$/EC:DMC (1:1 by vol.)) and as-prepared LiFSA/DMC solutions with various salt-to-solvent molar ratios upon heating was measured on a thermogravimetric analyzer (EXSTAR 6000, Seiko Instruments Inc.). The samples were firstly sealed in an Al pan in the glove box. A pinhole was punched to allow gas escape during the measurement just before the sample loading. Before heating up (5 °C min^{-1}), the loaded sample was hold at 33 °C for 20 mins in order to remove the air in the test chamber with the purge Ar gas (200 ml min^{-1}). During this period, a large weight loss was observed for the dilute electrolytes due to the volatilization of DMC solvent.

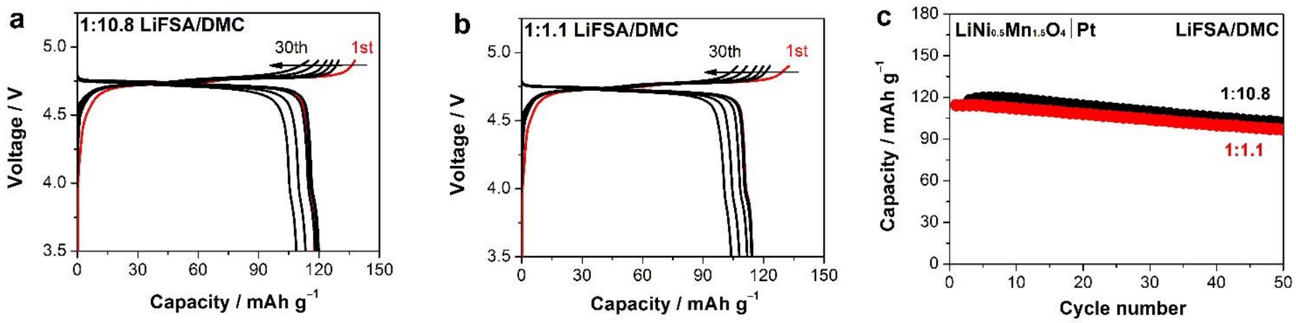

Supplementary Figure 2 Performance of $LiNi_{0.5}Mn_{1.5}O_4$ electrode (Pt current collector) in a three-electrode cell. Charge-discharge voltage curves of a three-electrode cell using (**a**) dilute 1:10.8 and (**b**) superconcentrated 1:1.1 LiFSA/DMC electrolytes. (**c**) Their discharge capacities (Li^+ intercalation) dependent of cycle number. Charge/discharge tests were conducted at 25 °C in a cutoff voltage of 3.5-4.9 V at a C/5 rate. 1C-rate corresponds to 147 mA g^{-1} on the weight basis of the $LiMi_{0.5}Mn_{1.5}O_4$ electrode. Using Pt as the current collector, both dilute and concentrated electrolytes enable highly reversible charge/discharge on the 5V-class $LiMi_{0.5}Mn_{1.5}O_4$ electrode without significant difference.

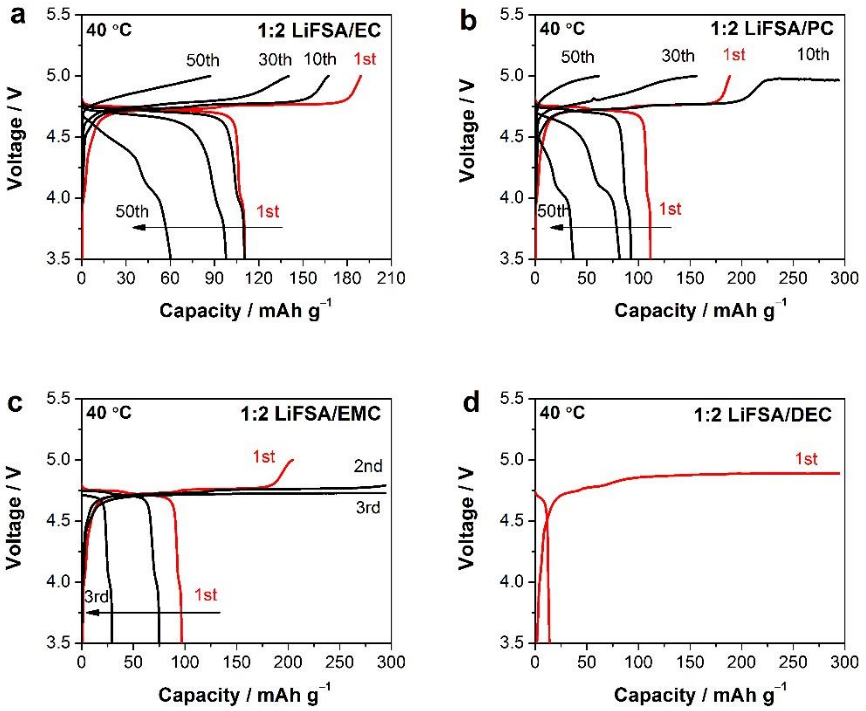

Supplementary Figure 3 Charge-discharge voltage curves of $LiNi_{0.5}Mn_{1.5}O_4$ electrode (Al current collector) in a half cell using various 1:2 LiFSA/carbonate electrolytes. The results indicate a salt-to-solvent molar ratio of 1:2 is not sufficient for an electrolyte to inhibit the anodic Al dissolution fully. Charge/discharge tests were conducted at 40 °C in a cutoff voltage of 3.5-5.0 V at a C/5 rate. 1C-rate corresponds to 147 mA g^{-1} on the weight basis of the $LiMi_{0.5}Mn_{1.5}O_4$ electrode.

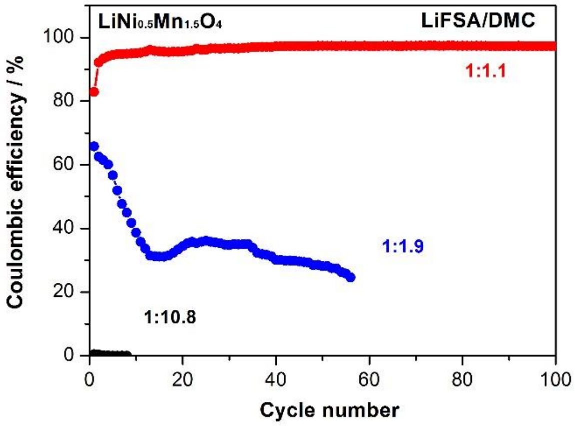

Supplementary Figure 4 Cyclic coulombic efficiency of the $LiNi_{0.5}Mn_{1.5}O_4$ electrode (Al current collector) in a half cell using various concentrations of LiFSA/DMC electrolytes. A charge/discharge test was conducted at a C/5 rate and 25 °C in a cutoff voltage of 3.5-5.2 V for 1:1.1 LiFSA/DMC electrolyte and a cutoff voltage of 3.5-4.9 V for 1:10.8 and 1:1.9 LiFSA/DMC electrolytes with a time restriction of 10 hours.

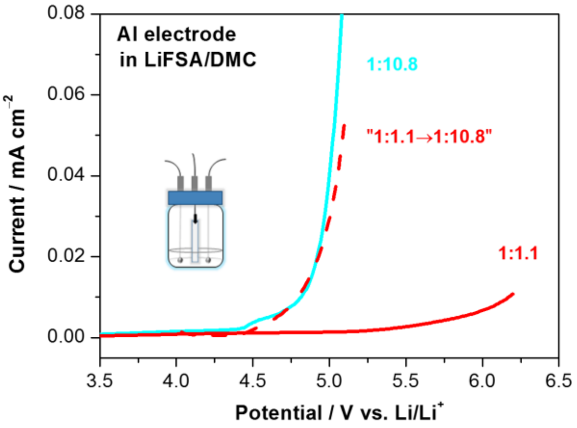

Supplementary Figure 5 Comparison of LSV results between fresh and polarized Al electrodes in the LiFSA/DMC electrolytes in a three-electrode beaker cell. The solid lines are the LSV results of fresh Al electrodes in dilute 1:10.8 and superconcentrated 1:1.1 LiFSA/DMC electrolytes. To judge if a more protective surface film is formed in the concentrated electrolytes, we firstly polarized an Al electrode in a beaker cell containing superconcentrated 1:1.1 LiFSA/DMC electrolyte; immediately after that we transferred this polarized Al electrode into another cell containing dilute 1:10.8 LiFSA/DMC electrolyte for a new LSV test (shown as the dash line). The result indicates no significant difference of the LSV profiles between the polarized and fresh Al electrodes ("1:1.1→1:10.8" vs. 1:10.8). This does not support the existence of a better surface film produced in the concentrated electrolyte. The scan rate is 1.0 mV s^{-1}. The use of beaker cell (inset) favors the exchange of electrolyte without damage of surface of a polarized Al electrode.

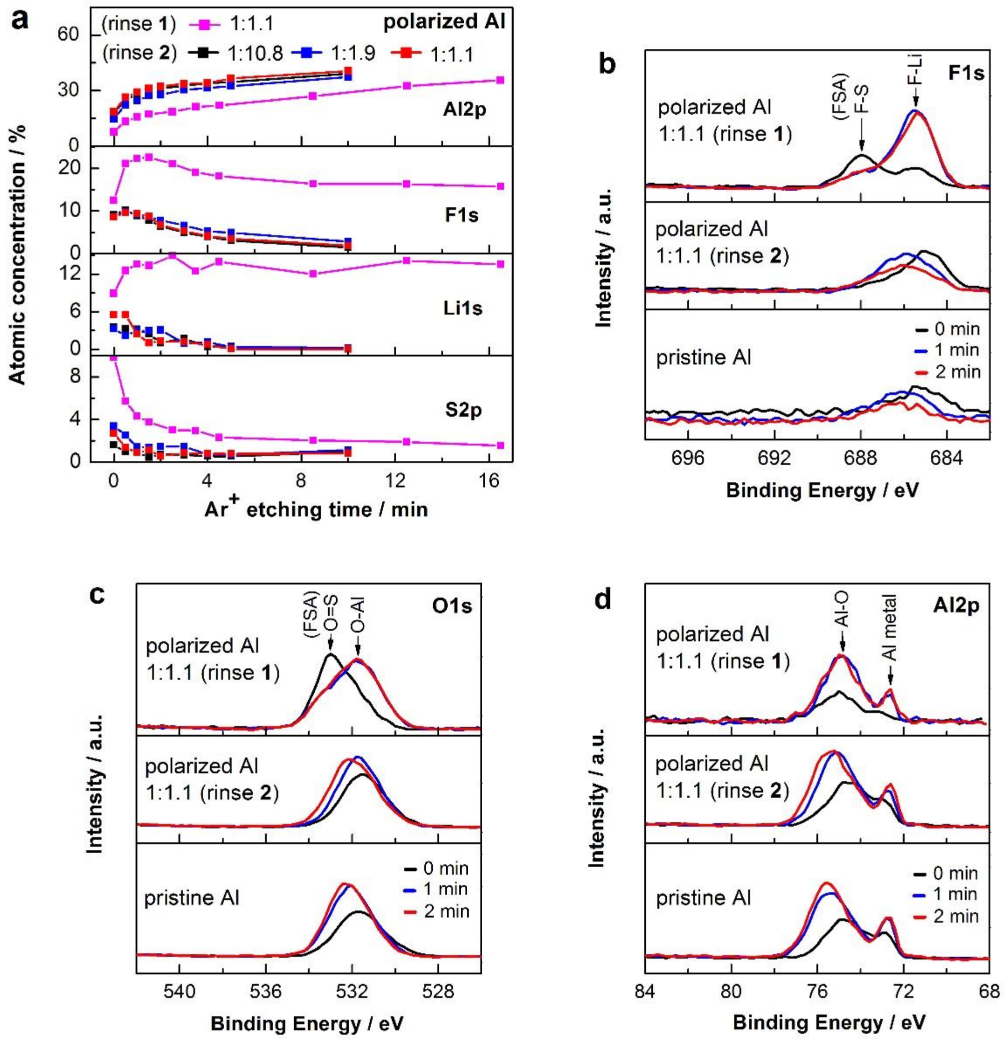

Supplementary Figure 6 XPS analysis of the polarized aluminum electrodes. (**a**) XPS depth profiles (500 V Ar^+ etching) of Al electrodes polarized in various salt-to-solvent molar ratios of LiFSA/DMC electrolytes and then treated by insufficient “rinse **1**” and sufficient “rinse **2**” (see **Supplementary Methods**). For the polarized Al electrodes with rinse **2**, the composition and thickness of the surface films showed independent of concentration of the used electrolytes. In contrast, a gloss of a thicker surface film of LiF appeared for the polarized Al electrode in the superconcentrated 1:1.1 LiFSA/DMC electrolyte with rinse **1**, which is actually caused by the decomposition of the unrinsed LiFSA left on the Al surface induced by Ar^+ etching (see Supplementary Fig. 7). (**b-d**) demonstrate F1s, O1s and Al2p spectra for the polarized and pristine Al electrodes upon 0, 1 and 2 mins of Ar^+ etching, respectively. Little difference on the surface can be distinguished between the polarized Al electrode (rinse **2**) and the pristine Al electrode. Whereas significant residual LiFSA was evidenced on the surface of the polarized Al electrode (rinse **1**) according to F1s and O1s spectra before Ar^+ etching, which are almost identical with those of pure LiFSA (see Supplementary Fig. 7).

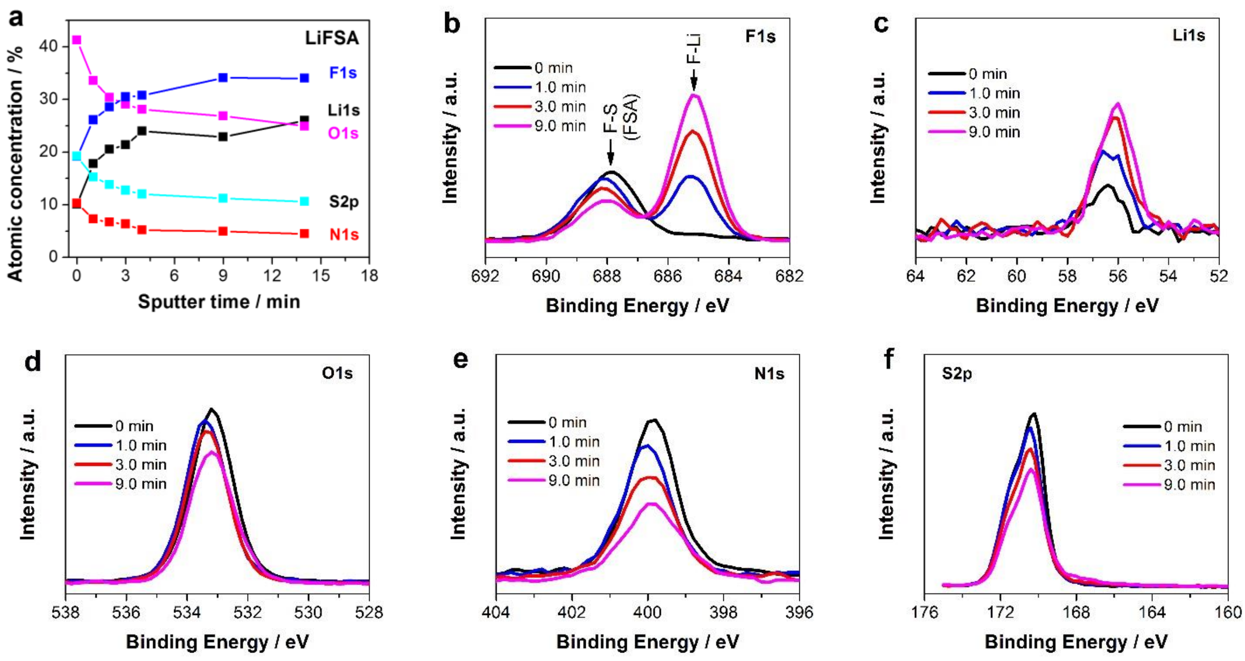

Supplementary Figure 7 XPS analysis of pure LiFSA upon Ar^+ etching. (**a**) XPS depth profiles (500 V Ar^+ etching) of pure LiFSA powder. (**b**-**f**) demonstrate the corresponding XPS spectra upon different time of Ar^+ etching. Before Ar^+ etching, the detected F : Li : O : N : S ratio of sample is 1 : 2 : 4 : 1 : 2, which is consistent of the stoichiometric composition of LiFSA. The F1s spectrum of LiFSA shows a single peak at ca. 688.0 eV. As Ar^+ etching, the intensity of this peak decreases while that of a new peak at 685.2 eV (corresponding to LiF) increases. Meanwhile, the intensity of Li1s spectrum increases and those of O1s, N1s and S2p spectra decreases. Those results suggest that LiFSA undergoes decomposition upon Ar^+ etching with generation of LiF and other products (containing N, O, S and F). In addition, the peak position and shape of O1s, N1s and S2p spectra have no significant change upon Ar^+ etching implying that almost no other species exist on the sample except from LiF and LiFSA. It is likely that the decomposed products besides LiF are volatile or easily peeled off by the Ar^+ beam. And thus, the concentrations of Li and F increase while those of O, N and S decrease upon Ar^+ etching.

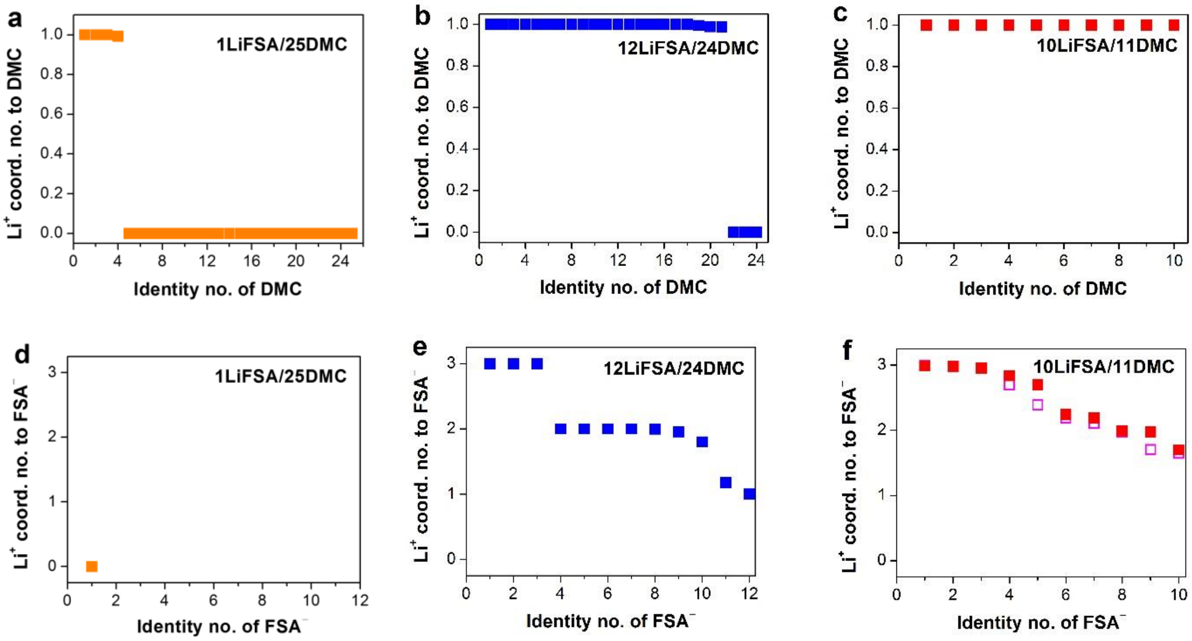

Supplementary Figure 8 Coordination environment of Li^+ to solvents and anions in different concentrations of solutions. (**a–c**) are Li^+ coordination numbers to DMC molecules; (**d–f**) are Li^+ coordination numbers to FSA^- anions. The coordination numbers are average vales during all DFT-MD simulation time of 10 ps (100,000 steps × 0.1 fs intervals). All DMC molecules and FSA^- anions are labeled with identity numbers. The coordination of Li^+−DMC and Li^+−FSA^- in the dilute and moderately concentrated solution is through the interaction between Li^+ and oxygen. In the superconcentrated solution, significant amount of nitrogen also participate in the Li^+−FSA^- coordination. The hollow data in (**f**) is the result with only consideration of oxygen coordinating to Li^+. The solid data in (**f**) is the result with consideration of both oxygen and nitrogen coordinating to Li^+.

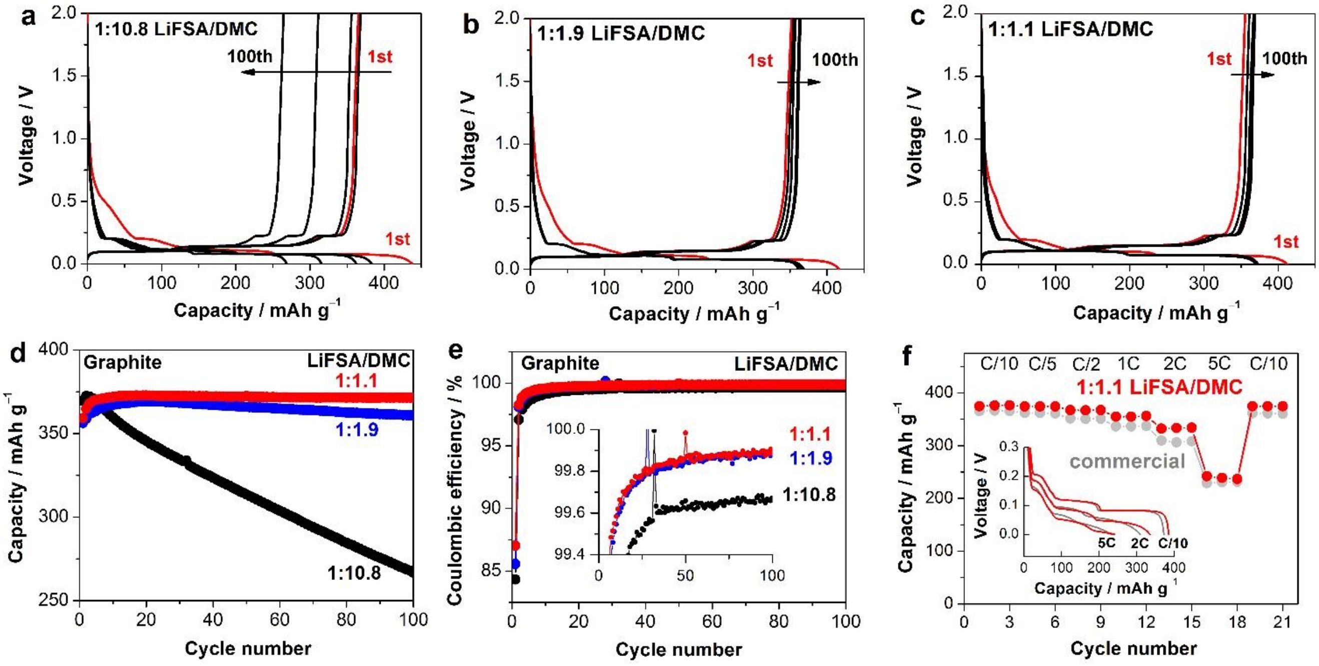

Supplementary Figure 9 Performance of the natural graphite electrode in a half-cell. Charge-discharge voltage curves of graphite│lithium metal half-cells using (**a**) dilute 1:10.8, (**b**) moderately concentrated 1:2, and (**c**) superconcentrated 1:1.1 LiFSA/DMC electrolytes at a C/5 rate. Curves of 1st, 2nd, 10th, 50th and 100th cycles are shown. (**d**) Discharge (Li^+ deintercalation) capacity retention and (**e**) coulombic efficiency of the half-cells at a C/5 rate. The inset in (**e**) is a magnified view. (**f**) Comparison of the rate capacity of the half cells using the commercial and superconcentrated LiFSA/DMC electrolytes with an inset of the representative charge profiles at different C-rates. Charge-discharge tests were conducted at 25 °C in a cutoff voltage of 0-2.5 V. 1C-rate corresponds to 372 mA g^{-1} on the weight basis of the graphite electrode.

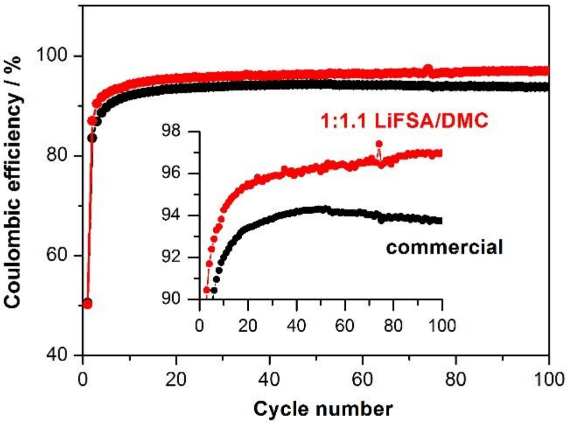

Supplementary Figure 10 Cyclic coulombic efficiency of a high-voltage $LiNi_{0.5}Mn_{1.5}O_4$│natural graphite battery using commercial and superconcentrated electrolytes. The charge-discharge cycling (3.5-4.8 V) was conducted at a C/5 rate and 40 °C. 1C-rate corresponds to 147 mA g^{-1} on the weight basis of the $LiMi_{0.5}Mn_{1.5}O_4$ electrode. The coulombic efficiency was not high under the present experimental condition. The seemingly low coulombic efficiency is sometimes understood on the basis of a shuttle mechanism, in which some oxidized/reduced species work as a redox shuttle to cause a parasitic reaction between the positive and negative electrodes. However, it should be noted that the seemingly low coulombic efficiency primarily resulted from our special cell design with a low mass loading on purpose to spotlight the Al corrosion, and is not an essential problem of the electrolytes. Because the cell components/active material ratio is quite high in this cell, even a slight amount of side reactions at cell components (other than active materials) will greatly lower the coulombic efficiency. Indeed, the coulombic efficiency is considerably improved by using a higher mass loading (~10 mg cm^{-2}), as shown in Supplementary Fig. 11.

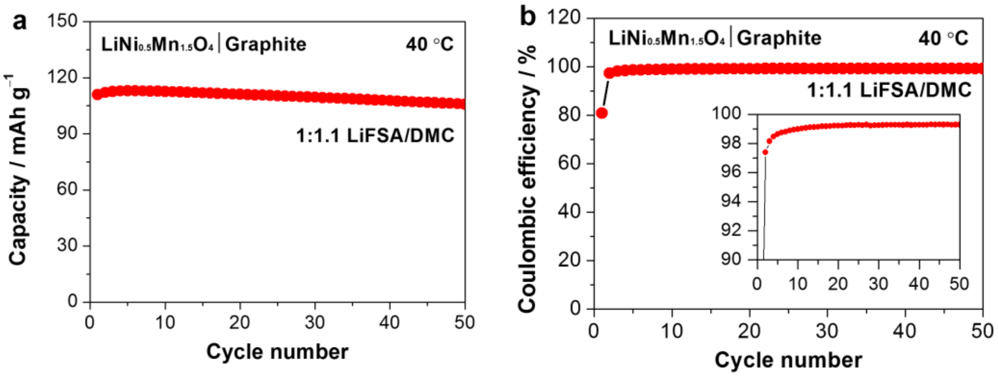

Supplementary Figure 11 Cycling performance of a high-voltage $LiNi_{0.5}Mn_{1.5}O_4$ | natural graphite battery (with a higher mass loading) using superconcentrated 1:1.1 LiFSA/DMC electrolyte. a) Discharge capacity retention and **b**) coulombic efficiency of the full cell at a C/5 rate and 40 °C. The mass loading of $LiNi_{0.5}Mn_{1.5}O_4$ and natural graphite is ~9.8 and ~3.4 mg cm^{-2}, respectively. Charge-discharge test was conducted in a cutoff voltage of 3.5-4.8 V. 1C-rate corresponds to 147 mA g^{-1} on the weight basis of the $LiMi_{0.5}Mn_{1.5}O_4$ electrode. Compared to the full cell with a low mass loading (Fig. 5 and Supplementary Fig. 10), the coulombic efficiency is significantly improved to well over 99 %.

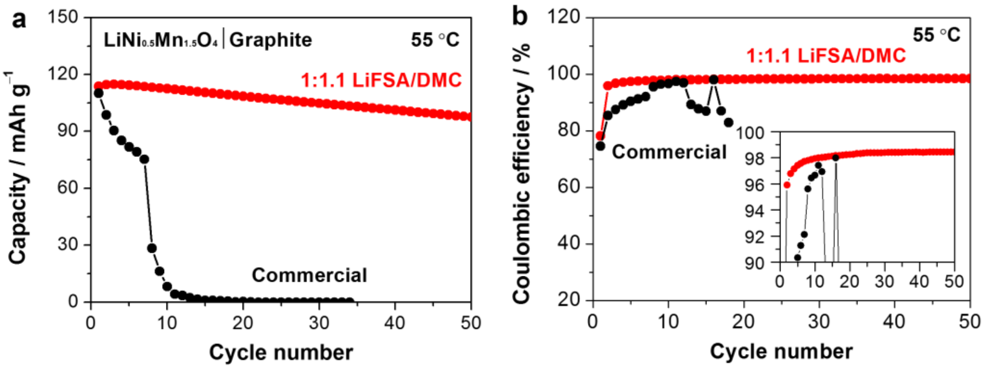

Supplementary Figure 12 High-temperature cycling performance of a high-voltage $LiNi_{0.5}Mn_{1.5}O_4$│natural graphite battery (with a higher mass loading). a) Discharge capacity retention and **b**) coulombic efficiency of the full cells with a commercial 1.0 mol dm^{-3} $LiPF_6$/EC:DMC (1:1 by vol.) electrolyte and a superconcentrated 1:1.1 LiFSA/DMC electrolyte at a C/5 rate and 55 °C. The mass loading of $LiNi_{0.5}Mn_{1.5}O_4$ and natural graphite is ~9.8 and ~3.4 mg cm^{-2}, respectively. Charge-discharge test was conducted in a cutoff voltage of 3.5-4.8 V. 1C-rate corresponds to 147 mA g^{-1} on the weight basis of the $LiMi_{0.5}Mn_{1.5}O_4$ electrode.

Supplementary Table 1 Physicochemical properties of LiFSA/carbonate esters solutions at 30 °C

Electrolyte	Molar ratio	Mole fraction	Concentration mol dm^{-3}	Density g cm^{-3}	Viscosity mPa s	Conductivity mS cm^{-1}
LiFSA/DMC	1:22.7	0.04	0.50	1.11	0.9	3.3
	1:10	0.09	1.08	1.18	1.5	9.9
	1:4.9	0.17	2.02	1.27	3.9	12.2
	1:2.9	0.26	3.04	1.36	12.9	8.1
	1:2.0	0.33	3.91	1.44	35.3	4.2
	1:1.3	0.43	5.03	1.53	124.2	1.7
	1:1.1	0.48	5.49	1.57	238.9	1.1
LiFSA/EC	1:22.7	0.04	0.63	1.38	4.1	8.7
	1:10	0.09	1.34	1.43	8.1	9.7
	1:4.9	0.17	2.43	1.50	33.1	5.6
	1:2.9	0.26	3.56	1.57	103.2	2.3
	1:2.0	0.33	4.50	1.63	219.8	1.5
	1:1.3	0.43	5.67	1.71	621.9	0.8
LiFSA/EC:DMC (1:1 by mol.)	1:22.7	0.04	0.56	1.24	1.9	11.5
	1:10	0.09	1.20	1.30	3.4	14.0
	1:4.9	0.17	2.21	1.38	9.8	9.8
	1:2.9	0.26	3.28	1.46	36.9	4.5
	1:2.0	0.33	4.18	1.53	86.2	2.4
	1:1.3	0.43	5.34	1.62	307.8	1.0

***Note**: Mole fraction is the molar amount of LiFSA salt divided by the total molar amount of salt and solvents. Mole concentration is the molar amount of LiFSA salt divided by the total volume of the solution.*

Supplementary Methods

X-ray photoemission spectroscopy

The polarized Al electrodes (diameter 1.6 cm) were made in the coin cells using various concentrations of LiFSA/DMC electrolytes and lithium metal as the counter electrode. After 4 cycles of charge/discharge at a constant current of 5 μA between 3.5~4.9 V, the cells were disassembled in the glove box. The obtained polarized Al electrodes were then subjected to one time of rinse (3.0 ml DMC for 30 seconds, marked as "rinse **1**") or two times of rinse (new 3.0 ml DMC for another 30 seconds, marked as "rinse **2**") and subsequent dryness. An "XPS Transfer Vessel" was used for sample transfer from the glove box to the XPS chamber avoiding samples contact to air and moisture. The surface analysis of samples was performed on an X-ray photoemission spectrometer (PHI 5000 VersaProbe, ULVAC-PHI Inc.) with a monochromatized Al Kα radiation (25 W, 15 kV). The analysis area is 100 × 100 μm. A charge neutralizer was applied to compensate the surface charge of sample. Ar^+ etching was carried out at 500 V to obtain the depth profiles. All spectra were calibrated to the metallic Al2p peak at 72.6 eV. Pristine Al electrode and pure LiFSA powder were also studied and used as references.
